# Preparation and Antifouling Property of Polyurethane Film Modified by PHMG and HA Using Layer-by-Layer Assembly

**DOI:** 10.3390/polym13060934

**Published:** 2021-03-18

**Authors:** Huihui Yuan, Chenli Xue, Jiaqian Zhu, Zhaogang Yang, Minbo Lan

**Affiliations:** 1 Shanghai Key Laboratory of Functional Materials Chemistry, School of Chemistry & Molecular Engineering, East China University of Science and Technology, Shanghai 200237, China; yuanhuihui@ecust.edu.cn (H.Y.); Y30180191@mail.ecust.edu.cn (C.X.); Y30190263@mail.ecust.edu.cn (J.Z.); 2 Department of Radiation Oncology, The University of Texas Southwestern Medical Center, Dallas, TX 75390, USA; zhaogang.yang@utsouthwestern.edu

**Keywords:** modified PU film, protein adsorption, antibacterial, hyaluronic acid (HA), polyhexamethylene guanidine (PHMG)

## Abstract

To reduce the possibility of bacterial infection and implant-related complications, surface modification on polyurethane (PU) film is an ideal solution to endow hydrophobic PU with antibacterial and antifouling properties. In this work, a variety of polyhexamethylene guanidine/ hyaluronic acid (PHMG/HA) multilayer films were self-assembled layer-by-layer on PU films using polyanions, carboxyl-activated HA, and polycations PHMG by controlling the concentration of these polyelectrolytes as well as the number of layers self-assembled. Attenuated total reflection Fourier transform infrared spectroscopy (ATR-FTIR) spectra, water contact angle (WCA), and A Atomic force microscope (AFM) of PU and modified PU films were studied. Protein adsorption and bacterial adhesion as well as the cytotoxicity against L929 of the film on selected PU-(PHMG/HA)_5_/5-5 were estimated. The results showed that PU-(PHMG/HA)_5_/5-5 had the best hydrophilicity among all the prepared films, possessing the lowest level of protein adsorption. Meanwhile, this film showed efficient broad-spectrum antibacterial performance as well as significant resistance of bacterial adhesion of more than a 99.9% drop for the selected bacteria. Moreover, almost no influence on cell viability of L929 enhanced the biocompatibility of film. Therefore, the modified PU films with admirable protein absorption resistance, antimicrobial performance, and biocompatibility would have promising applications in biomedical aspect.

## 1. Introduction

Polyurethane (PU) ureteral stents are wildly used in urological clinics for their good flexibility and elasticity, biocompatibility, and low cost compared to what or within which range of materials [1,2]. However, the hydrophobic surface of PU reduces the antifouling and antimicrobial properties, which results in the increasing amount of protein adsorption, bacteria adhesion, and salt deposition in a urine environment [3,4]. Thus, encrustation, infection, and implant-related post-complications such as ureteral stricture, perforation, and mucosal injury [5,6] are observed during the implantation in vivo. Thus, the antimicrobial property of the stent’s surface is vital for its service life in clinic. It is believed that an ideal antibacterial surface possesses properties of repelling protein adsorption in order to prevent initial bacteria attachment [7,8], repelling direct bacteria adhesion and killing the attached bacteria during the period of implantation in human body. With the aim of achieving these targets, various materials are applied to modify the PU surface to improve its hydrophilicity or to confer its antibacterial properties. Yuan et al. [9] modified chondroitin sulfate onto the PU surface to improve its hydrophilicity and reduce the protein adsorption. Manohar et al. [4] covalently crosslinked papain onto PU to prevent bacterial adhesion. Fischer et al. [10] attached a hydrogel coating loaded with Ag nanoparticles to a PU conduit to improve its antibacterial activity. However most material modifications can only improve one aspect of performance of the surface. Therefore, modification with an antibacterial agent and antifouling material on the surface is a good strategy to endow the PU surface with both antifouling properties and bactericidal properties [11,12], which would be a desirable antibacterial surface for clinical usage.

A surface with good hydrophilicity has been proven to effectively prevent non-specific protein adsorption. Hyaluronic acid (HA) is one of the most hydrophilic molecules in nature with non-toxic, non-immunogenic, non-inflammatory, and biodegradable properties [13,14,15,16]. HA is also a polyanion glycosaminoglycan that can repel most negatively-charged proteins and bacteria with negatively-charged cell membranes by electrostatic repulsive force. Conversely, it would be able to electrostatically attract cationic antimicrobial such as chitosan [17,18], quaternary ammonium salts [19,20], and cationic antimicrobial peptides [21] to integrate antibacterial function. Hence, HA is suitable for the surface modification of materials to reach the ultimate purpose of reducing bacterial adhesion [11,22]. Polyhexamethylene guanidine (PHMG) is a highly water-soluble, colorless, and odorless positively-charged antimicrobial [23]. Due to its broad spectrum activity against bacteria and fungi [24,25] and its low toxicity to mammals [26], PHMG has been successfully applied in several products such as topical wound solutions, contact lens cleaning products, and cosmetics [26,27,28]. Wei et al. [29] demonstrated that aqueous solutions of PHMG with concentrations as low as 1.0 ppm showed more than a 90.0% antibacterial rate. Ding et al. [30] bonded PHMG to resins to generate antibacterial acrylic coatings. The inhibitory factors against both *Escherichia coli* (*E. coli*) and *Staphylococcus aureus* (*S. aureus*) were over 99.99% at a PHMG content of 1.0 wt%. Therefore, the combination of HA and PHMG provides the modified surface with the desirable multifunction of hydrophilicity and antibacterial activity.

In this study, we created PHMG/HA multilayer films on PU by using layer-by-layer self-assembly with HA and PHMG as polyanions and polycations to render the surface of PU films with both antifouling and antibacterial properties. PHMG was chemically bonded to the PU surface via the reaction between amide groups of PHMG and isocyanato groups modified on PU films. Negatively-charged HA was assembled by electrostatic adsorption with positively-charged PHMG modified on PU films. Simultaneously, HA and PHMG were also covalently combined by the reaction of the partial activated carboxyl group of HA and amide groups of PHMG. The different concentrations of HA and PHMG as well as and the number of assembled layers were studied to attain PU-(PHMG/HA)_n_ films with different properties. The surface properties of modified and unmodified PU films were characterized by attenuated total reflection Fourier transform infrared spectroscopy (ATR-FTIR), water contact angle (WCA), and atomic force microscopy (AFM). The antifouling and antibacterial properties of the surface were detected by a bicinchoninic acid (BCA) protein detection kit and bacterial assay. Finally, the cytotoxicity of L929 cells was estimated for the improvement in the biocompatibility of the material.

## 2. Materials and Methods

### 2.1. Materials

PU (pellethane 2363-80AE) was provided by Lubrizol Corporation (Wickliffe, OH, USA). Methylenediphenyl 4,4′-Diisocyanate (MDI) was bought from Aladdin Chemical Co. Ltd. (Shanghai, China). N,N-dimethylformamide (DMF), dimethyl sulfoxide (DMSO), toluene, and triethylamine were purchased from Sinopharm Chemical Reagent Co. Ltd. (Shanghai, China), and HA was obtained from Xianding Biotechnology Co. Ltd. (Shanghai, China). 1-(3-Dimethylaminopropyl)-3-ethylcarbodiimide hydrochloride (EDC∙HCl), N-Hydroxysuccinimide (NHS), Lysozyme (LYS), bovine fibrinogen (BFG), human serum albumin (HSA), and 3-(4,5-dimethyl-2-thiazolyl)-2,5-diphenyl-2-H-tetrazolium bromide (MTT) were obtained from Yuanye Bio-Technology Co. Ltd. (Shanghai, China). Sodium dodecyl sulfate (SDS) and the Micro BCA Protein Assay Kit were obtained from Sangon Biotech (Shanghai, China) Co. Ltd. Fetal bovine serum (FBS) and Dulbecco’s modified Eagle medium (DMEM) medium were obtained from Gibco (Grand Island, NY, USA). *E. coli* (DH5 alpha), *S. aureus* (ATCC 6538), and *P. aeruginosa* (ATCC 2785) and lysogenic broth (LB) medium were provided by Professor Cui, East China University of Science and Technology (ECUST, Shanghai, China). PHMG was provided by Professor Guan, ECUST.

### 2.2. The Fabrication of the PU-(PHMG/HA)_n_ Films

Ten grams of commercial PU was dissolved in 100 mL DMF by magnetic stirring. Then, the PU solution was vacuum dried at 60 °C for 72 h to obtain thin PU films, and the films were cut into discs (6 mm in diameter and 1 mm in thickness). Afterward, PU films were put into MDI solution for a 3 h reaction to obtain PU-NCO films. Finally, the PU-PHMG film was prepared by the reaction of amino groups of PHMG with PU-NCO.

Carboxyl activated HA intermediate was prepared according to the literature [31]. A total of 0.24 g EDC∙HCl and 0.15 g NHS were slowly added to 50 mL HA solution (1%, m/v) in turn under the condition of pH 4.75 with stirring for a 2.5 h reaction. After that, the reaction was terminated by increasing the pH value to 7.5. The reaction product was further dialyzed to remove EDC and NHS at room temperature for two days. Finally, carboxyl-activated HA was obtained and freeze-dried by a vacuum freeze dryer.

**Figure 1:**
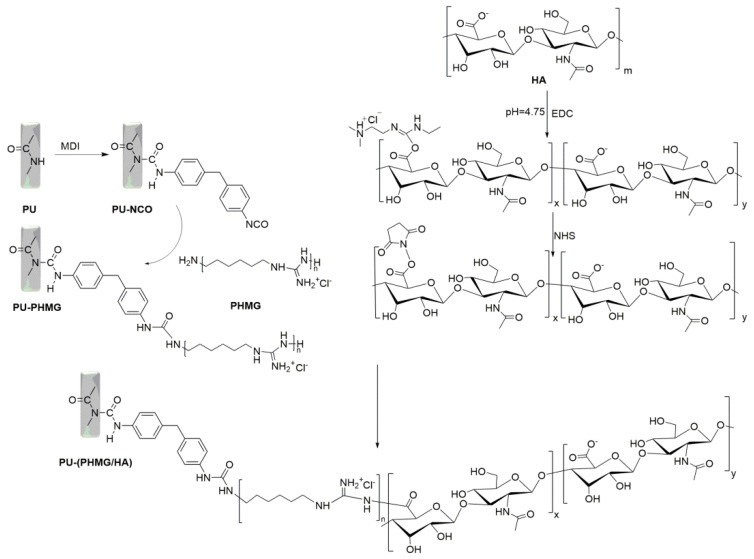
Synthesis procedure for the PU-(PHMG/HA) films.

PU-(PHMG/HA)_n_ films were prepared by sequential assembling HA and PHMG onto the PU-PHMG film layer by layer. Figure 1 illustrates the fabrication process of the PU-(PHMG/HA) films. The assembling time per polymer layer was 20 min [32]. In between the PHMG and HA assembling steps, the assembled films were put into deionized (DI) water to remove the unassembled molecules and dried at 60 °C. Different samples were obtained by adjusting the polyelectrolyte concentration and the number of bilayers self-assembled, and Table 1 lists the formulations for various samples.

### 2.3. Characterizations of the Films

The surface chemical structure of the film was identified by a FTIR Spectrometer (Thermo Nicolet 6700, Madison, WI, USA) with an ATR device. The spectra were collected at the following instrument parameters: scan range 400–4000 cm^−1^, resolution 4 cm^−1^, and scan times 16 [33]. The WCA of the surface was measured by a contact angle meter (Powereach JC 2000D, Shanghai, China). The surface morphology of the films was analyzed by AFM (Veeco DI3100, Plainview, NY, USA). The surface roughness of the films was the average roughness of three areas.

### 2.4. Protein Adsorption

BFG, HSA, and LYS were selected for protein adsorption experiments to investigate the anti-protein adsorption properties of the films. The films were soaked in 1 mL protein solution (1 mg/mL) and incubated at 37 °C for 1 h, and then washed with PBS buffer solution (pH 7.4) and ultrapure water in order to remove the unabsorbed proteins on the surface. A total of 0.5 mL SDS (1% *w*/*v*) solution was used to elute the adsorbed proteins and incubated with films at 37 °C for 2 h. The amount of protein was calculated by measuring the absorbance of the eluate at 562 nm with a UV–Vis spectrophotometer (Thermo Fisher Evolution 220, Waltham, MA, USA) using the BCA assay.

### 2.5. Bacteria Adhesion

Gram-positive bacteria (*S. aureus*) and Gram-negative bacteria (*E. coli* and *P. aeruginosa*) were selected to test the antibacterial activity of the films. A single pure colony was cultured in LB medium at 37 °C for 18 h. The supernatant was removed by centrifugation and the bacteria were diluted to 10^8^ CFU/mL with PBS. The films were immersed in 1 mL bacterial solution and incubated at 37 °C with shaking for 24 h. Nonadherent bacteria on the film surface were removed by washing with PBS three times, and then the adherent bacteria were eluted into 1 mL PBS by sonication for 10 min. The amount of bacteria was measured by the flat colony counting method. Additionally, the adherent bacteria on film were studied by confocal laser scanning microscopy (CLSM, NIKON A1R, Tokyo, Japan) after fluorescein diacetate (FDA)/propidium iodide (PI) staining. PI stain selectively binds to the dead bacteria and stains them fluorescent red, whereas the FDA stain selectively binds to the live bacteria and stains them fluorescent green during CLSM imaging.

### 2.6. Cytotoxicity Test

Cytotoxicity of films was determined by the MTT assay [34,35]. The conditioned cell culture medium was obtained by immersing films in 1 mL cell culture medium, which was applied to assay the effect of the films on cultured cells. L929 cells were seeded into 96-well plates (7000 cells per well) and cultured in an incubator with 5% CO_2_ at 37 °C for 24 h before they were incubated with 200 μL conditioned cell culture medium for 24 h. A total of 200 μL MTT reagent was added to each well for a further 4 h incubation at 37 °C. Then, the formazan precipitate was extracted by 150 μL DMSO, and the absorbance (492 nm) was recorded using a Multiskan MK3 ELISA reader (Thermo Fisher, Waltham, MA, USA).

### 2.7. Statistical Analysis

Statistical analyses were performed by SPSS for Windows software, version 18 (SPSS, Chicago, IL, USA). Data are presented as mean ± standard deviations (SD) of at least three replicates. *p* value < 0.05 was considered statistically significant.

## 3. Results and Discussion

### 3.1. Characterization of the Films

**Figure 2:**
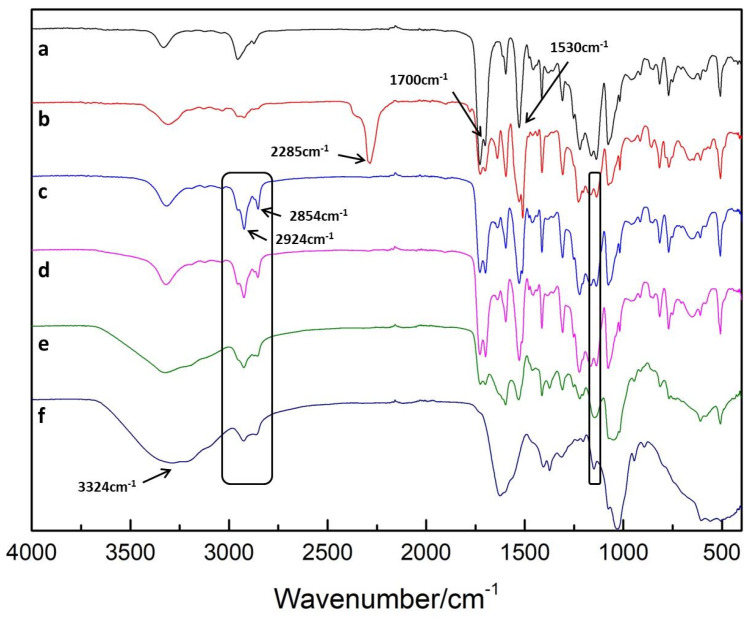
ATR–FTIR spectra of (**a**) PU; (**b**)PU-NCO; (**c**)PU-PHMG; (**d**) PU-(PHMG/HA)_1_/5-5; (**e**) PU-(PHMG/HA)_5_/5-5; (**f**) PU-(PHMG/HA)_10_/5-5.

The ATR-FTIR spectra of films at different preparation stages are shown in Figure 2. PU presented peaks originating from C=O and C–N at 1700 cm^−1^ and 1530 cm^−1^, respectively (Figure 2a) [36]. A dominant absorption peak was observed at 2285 cm^−1^, which suggested that the –NCO group was successfully grafted on the PU surface (Figure 2b) [37,38]. However, Figure 2c shows that the peak of –NCO disappeared, and two symmetric and asymmetric –CH_2_ stretching vibrations attributed to PHMG were noted at 2854 cm^−1^ and 2924 cm^−1^ [39], respectively, which confirmed that –NCO totally reacted with the –NH_2_ of PHMG. Nevertheless, the spectra of PU-(PHMG/HA) (Figure 2d) showed no obvious change compared with that of PU-PHMG, suggesting that the first layer of HA might have little influence on the improvement of the surface properties. The wide peaks at 3324 cm^−1^ assigned to the –OH group in HA increased (Figure 2e,f), indicating that the PHMG/HA bilayers were successfully assembled on the PU film [40]. Furthermore, the relatively broad peak at 1150 cm^−1^ belonging to the ester group [41] in COOH– activated HA was found in the spectra of PU-(PHMG/HA)_5_/5-5 and PU-(PHMG/HA)_10_/5-5, while it did not appear in that of PU-PHMG and PU-(PHMG/HA)_1_/5-5. This phenomenon indicated that HA partially covalently bonded on the surface as expected. The reason might be due to the following: The activated –COOH provided by HA was not sufficient and completely reacted with the –NH_2_ of PU-PHMG to –CO–NH during the preparation of the first bilayer PHMG/HA. Additionally, the –CO–NH was not able to be distinguished due to its original existence in any of the PU and modified PU films. With the increase in bilayer number, more HA provided more reactive ester groups, which could meet the demand in crosslinking of HA-PHMG. Nevertheless, the peak of the ester group was reduced with the increased bilayer, according to the spectra comparison of PU-(PHMG/HA)_5_/5-5 and PU-(PHMG/HA)_10_/5-5. This might be attributed to molecular rearrangement during the proceeding of assembly, which created more chances for the ester group to react with –NH_2_. In addition, the peak of –CH_2_ weakened with the increased number of bilayers, but still existed on the surface of all assembled films in Figure 2d–f. It was supposed that the molecules of HA and PHMG were assembled in an entangled manner, which resulted in incomplete coverage of the HA chains on the surface [42].

**Figure 3:**
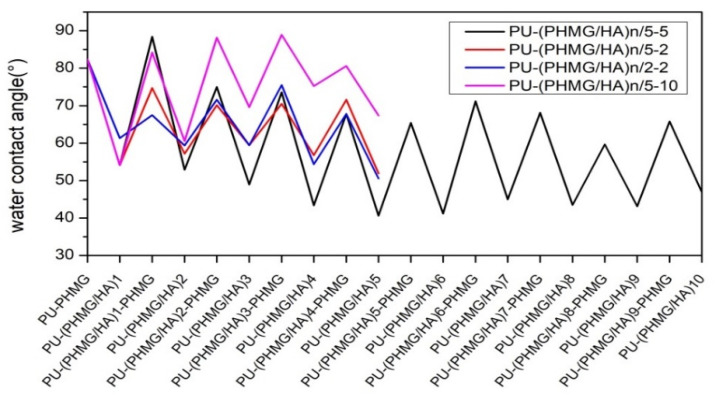
Water contact angle (WCA) of modified PU films. n in PU-(PHMG/HA)_n_/5-5, PU-(PHMG/HA)_n_/5-2, PU-(PHMG/HA)_n_/2-5 and PU-(PHMG/HA)_n_/5-10 was the number of bilayer.

The variation of the WCA was likewise related to the introduction of functional groups/molecules onto the surface. The WCA of the original PU was 90.1° due to its hydrophobicity [43,44]. The successful grafting of hydrophobic isocyanate on PU resulted in the WCA of PU-NCO increasing to 96.8° [37,45]. However, the succeeding PHMG onto the surface led to a low WCA (82.3°) of PU-PHMG because of the introduction of the hydrophilic –NH_2_ group. After HA was covalently bonded and electrostatic self-assembled onto PU-PHMG films, the surface became more hydrophilic. Subsequently, PHMG and HA alternately assembled onto the surface, which contributed to the WCA of corresponding films with a zig-zag effect (Figure 3). The HA (odd) layer achieved smaller WCA than that of the PHMG layer (even), suggesting higher hydrophilicity of HA than PHMG and proving that films with alternating deposition of polyelectrolyte were successfully obtained. In addition, the concentration of polyelectrolyte had an obvious effect on the WCA of the films. The increase or decrease in PHMG concentration both caused the WCA of the surface with relatively high value based on the comparison of preparation groups PHMG (10 mg/mL), PHMG (5 mg/mL), and PHMG (2 mg/mL) when HA was fixed at 5 mg/mL (Figure 3). One explanation might be less PHMG, leading to less HA loaded. The other might be the excessive PHMG providing more –CH_2_ exposed on the surface when they entangled with HA. Therefore, it was found that the combination of HA (5 mg/mL)-PHMG (5 mg/mL) achieved the greatest reduction in the WCA of the modified films among those prepared by other concentration combination of HA-PHMG. At this HA-PHMG concentration pair, HA and PHMG were well matched and reached a dynamic balance during the assembly process. Nevertheless, the WCA of PU-(PHMG/HA)_n_/5-5 had almost no apparent reduction, indicating that excessive assembled layers might have little impact on the function promotion of the surface.

**Figure 4:**
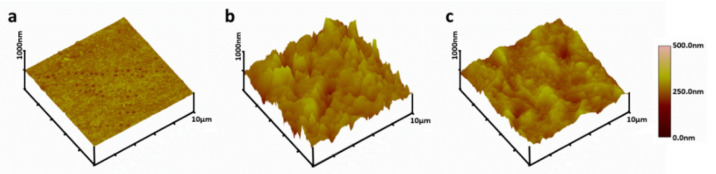
Atomic force microscopy (AFM) images of (**a**) PU; (**b**) PU-PHMG; (**c**) PU-(PHMG/HA)_5_/5-5.

The surface topography of the film was determined by AFM. The surface of the original PU was fairly flat and smooth with a root-mean-square (RMS) roughness of 36.4 ± 2.5 nm (Figure 4). However, the RMS of PU-PHMG surfaces increased significantly to 177.7 ± 2.3 nm (*p* < 0.001) compared to the PU films. One layer of HA assembled on PU-PHMG made little contribution to lower roughness of surface (173.9 ± 3.3 nm), which was consistent to the result of the ATR-FTIR spectra. However, after alternating PHMG and HA modification on PU films a few times, the surface roughness of PU-(PHMG/HA)_n_ (e.g., PU-(PHMG/HA)_5_/5-5) decreased in comparison with that of PU-PHMG, but was still rougher than that of PU. Table 2 lists the surface roughness value for various samples. With the increase of HA concentration, the roughness of the films showed no obvious change based on the comparison of PU-(PHMG/HA)_5_/2-2 and PU-(PHMG/HA)_5_/5-2. With the increase in PHMG concentration, the roughness of the films decreased in comparison with PU-(PHMG/HA)_5_/5-2, PU-(PHMG/HA)_5_/5-5, and PU-(PHMG/HA)_5_/5-10. This influence might be related to the molecular weight of HA (>10 kDa) and PHMG (~600 Da). PHMG with far lower molecular weight than HA had relative flexibility and more PHMG was able to fill the void, which resulted in the lower roughness of the surface. Additionally, the number of assembled layers positively influenced the roughness of the modified films at the fixed preparation concentration based on the comparison of PU-(PHMG/HA)_1_/5-5, PU-(PHMG/HA)_5_/5-5, and PU-(PHMG/HA)_10_/5-5, whereas the increase in the bilayer number had a minor contribution to lower the roughness when the number of bilayers was more than five. PU-(PHMG/HA)_10_/5-5 possessed the smoothest surface with a RMS roughness value of 130.8 ± 2.6 nm, followed by PU-(PHMG/HA)_5_/5-10 and PU-(PHMG/HA)_5_/5-5. The roughness of the above three films had no remarkable differences. Therefore, PU-(PHMG/HA)_5_/5-5 was the optimum film when taking into account the preparation costs.

### 3.2. Protein Adsorption

Adsorption of protein on the surface works as the initial step of biofouling when implanted in vivo and further compromises the surface properties, promotes cell attachment, and initiates the final foreign body response [46,47]. Thus, the resistance to protein adsorption of PU films is first taken into consideration.

Due to the inherent hydrophobic and electrostatic interactions between the surface and proteins, generally, the adsorption of proteins on the hydrophobic surface of PU films has no obvious inhibitory effect on the adhesion of any kind of contaminant [48,49]. Therefore, improving hydrophilicity is a valid approach to enhance the antifouling property of film to some extent.

**Figure 5:**
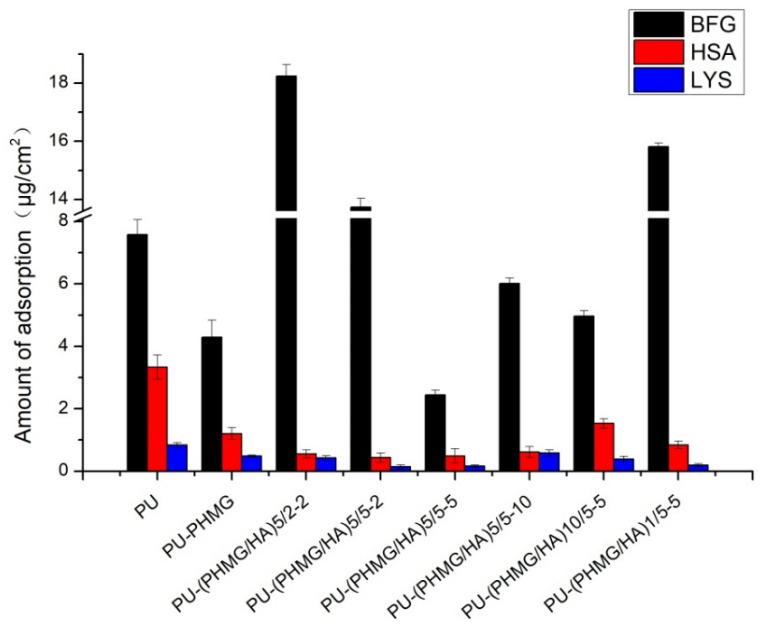
Protein adsorption on films.

The adsorption amount of BFG, HSA, and LYS on different films is listed in Figure 5. The PU-(PHMG/HA)_5_/5-5 surface exhibited the best resistance to protein adsorption, on which the adsorption levels of BFG, HSA and LYS were 2.43, 0.49, 0.16 µg/cm^2^, respectively. The adsorption amount followed the order of the molecular weight of proteins, that is BFG > HSA > LYS, as the high molecular weight resulted in the high amount of protein adsorbed on to patch at the same adsorption sites. The corresponding protein adsorption level was reduced 67.85%, 85.33%, and 80.31% compared with that on PU film. This is attributed to the higher hydrophilicity and lower surface roughness of PU-(PHMG/HA)_5_/5-5.

Besides hydrophilicity and surface roughness, the nanotopography structure also influenced the amount of protein adsorbed on these modified PU films. In Figure 5, BFG adsorption level on PU-PHMG films was markedly lower than that on PU-(PHMG/HA)_5_/2-2, PU-(PHMG/HA)_5_/5-2, and PU-(PHMG/HA)_1_/5-5, though the hydrophilicity and roughness of PU-PHMG were higher than that of the three films. The possible reason might be the brush structure of PHMG on PU films repulsing parts of the proteins. The surface entropy of PU-PHMG increased when PHMG brushes were compressed by BFG, which was disadvantageous to thermodynamic stability and led to repelling BFG adsorption on the surface [50,51]. Similarly, the amount of HSA and LYS adsorbed on PU-PHMG were comparatively low in comparison with that on PU-(PHMG/HA)_10_/5-5 and PU-(PHMG/HA)_5_/5-10, respectively. Thus, the brush structure dominated the protein adsorption on PU-PHMG. Likewise, the anomalous observation was the relatively low level of BFG adsorption on PU in contrast with that on films with high roughness and medium hydrophilicity. This result might embody the importance of the protein sharp in adsorption. Fibrinogen is known as a cylinder (diameter = 6 nm, length = 45 nm) [52], where the side-on orientations were difficult to adsorb stably on PU with a roughness of 36.4 nm because of stereohindrance. In addition, more assembled layers negatively affected the protein repelling property (typical case illustrated in PU-(PHMG/HA)_10_/5-5), which provided more internal space to capture smaller sized proteins such as HSA into the swelling inner films through the microstructure [52]. Among the three model proteins, LYS possessed the smallest size, and was a positively-charged (isoelectric point at 11.0) and ‘hard’ one. The electrostatic repulsion between LYS and PHMG was noticeable, which reflected in lower LYS adsorption quantity on the PU-(PHMG/HA)_1_/5-5 with high roughness and medium hydrophilicity compared with other PU-(PHMG/HA)_n_ films. After the formation of the first bilayer of PHMG and HA, the two molecules might be coiled and the positive charge of PHMG was not well covered by HA, as explained in th ATR-FTIR spectra, which resulted in a surface with good LYS repelling performance. As seen in the results above, the hydrophilicity, roughness, charges, and nanotopography structure of the surface as well as the size, shape, and charges of the proteins were the important factors for the protein adsorption property. Among these facts, surface hydrophilicity was a dominating one for protein adsorption.

### 3.3. Bacteria Adhesion

Bacterial infection is the main complication after stent implantation, and the adhesion and colonization of bacteria on the stent play an essential part in scaling. Therefore, antibacterial functionalization becomes the key target for surface modification. Three typical uropathogens were selected to evaluate the broad-spectrum antimicrobial properties of PU, PU-PHMG, and PU-(PHMG/HA)_5_/5-5 (best resistance to protein adsorption). The results are shown in Table 3. The amount of bacteria adhered to naked PU was 29.2 × 10^5^ CFU/cm^2^ for *E. coli*, 14.0 × 10^5^ CFU/cm^2^ for *P. aeruginosa*, and 24.3 × 10^5^ CFU/cm^2^ for *S. aureus*. Compared with PU, PU-PHMG showed excellent antibacterial effect against the three strains, and the adherence levels of the corresponding bacteria were as low as 0.0345 × 10^5^, 0.0153 × 10^5^, and 0.160 × 10^5^ CFU/cm^2^ with inhibitory rates of 99.88%, 99.89%, and 99.34%, respectively. The effective antibacterial activity of PU-PHMG was attributed to the bactericidal capacity of PHMG. The interaction between PHMG and the anionic components of bacterial cell wall compromises membrane integrity, further causing cell membrane rupture and leads to microbial death [53,54]. The inhibition rates of PU-(PHMG/HA)_5_/5-5 on *E. coli*, *P. aeruginosa*, and *S. aureus* were 99.99%, 99.96%, and 99.99%, respectively, indicating that the film had outstanding antibacterial activity. The inhibition rate was slightly higher than that of PU-PHMG, indicating that the improvement of surface hydrophilicity and roughness also affect the antibacterial effect.

**Figure 6:**
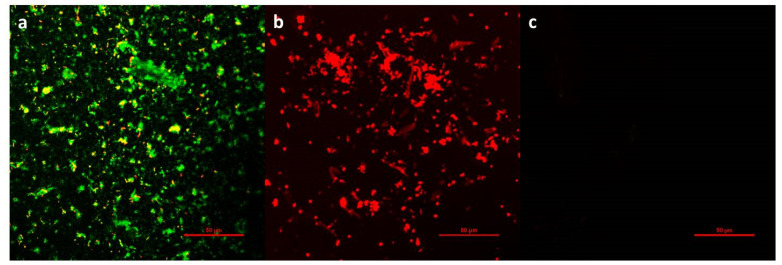
Confocal laser scanning microscopy (CLSM) images of *E. coli* adherent onto (**a**) PU, (**b**) PU-PHMG, and (**c**) PU-(PHMG/HA)_5_/5-5 film. The live bacteria appear as green fluorescence and dead bacteria appear as red fluorescence. Scale bars are 50 μm.

Aside from its antimicrobial activities, biofouling resistance is another crucial element affecting the long-term property of the films. Generally, bacteria will both adhere to the film to form colonies and participate in the formation of subsequent biofilms, covering up the function of antibacterial substances, and subsequently causing inevitable biological contamination. After incubation with bacteria for one day, the antifouling ability of the films was assessed by imaging bacterial adhesion on the surface. Figure 6 illustrates the bacterial adhesion on PU, PU-PHMG, and PU-(PHMG/HA)_5_/5-5, respectively. As observed, most of the live bacteria and few dead bacteria accumulated on the PU surface (Figure 6a) because of its hydrophobic property. PU-PHMG, in contrast, adhered to most of the dead bacteria (Figure 6b), showing that it had efficient antibacterial property but nearly no antifouling performance due to electrostatic adsorption and hydrophobic interaction [55]. Therefore, PU-PHMG merely maintained the antibacterial properties at the initial stage, but was gradually covered by dead bacteria and lost its function during long-term incubation with bacteria. To our delight, bacteria were barely observed on the surface of PU-(PHMG/HA)_5_/5-5 (Figure 6c), indicating no biofilm had formed. The high bactericidal efficiency was attributed to two aspects. On one hand, PHMG can kill bacteria temporarily adhered to the surface. On the other hand, the size of almost all of the bacteria was larger than 500 nm, which made the bacteria unable to be entrapped in the rough area. Therefore, the killed bacteria, gently adsorbed on the surface, can be easily stripped by simple hydraulic turbulence [46] due to the hydrophilicity of HA. The result of the antifouling property of three test films indicated that the antifouling property of the surface was important for an antibacterial effect. PU-(PHMG/HA)_5_/5-5 with good hydrophilicity containing PHMG and HA exhibited excellent antibacterial and antifouling properties, suggesting that it had an ideal antibacterial surface for future biomedical usage.

### 3.4. Cytotoxicity Test

**Figure 7:**
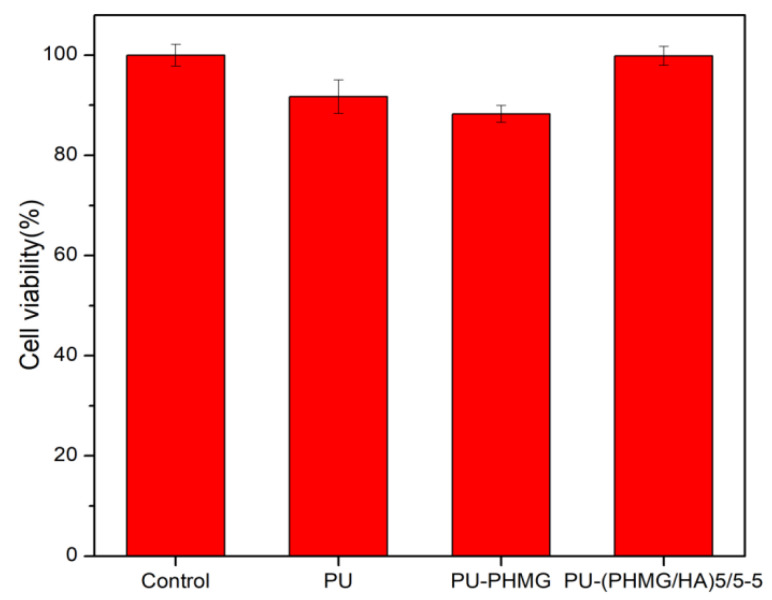
Cell viability of L929 after being cultured in PU, PU-PHMG, and PU-(PHMG/HA)_5_/5-5 extracts.

Biocompatibility is an essential requirement in bio-materials for their potential biomedical application [56]. Cytotoxicity testing can generally be performed in two ways—contact (direct) and extraction (indirect) [35]. The extraction method was applied due to the anti-adhesion property of the film surface, which was difficult for cells to adhere on. The conditioned cell culture medium mimicked the effect of the film on the physiological environment. The results of the cytotoxicity of L929 cultured in leaching solution of films are shown in Figure 7. PU and PU-PHMG films had high cell viability (over 88%) and the PU-(PHMG/HA)_5_/5-5 film had no cytotoxicity against L929 cell compared to the control, which indicated that the final surface modification was favorable to cell viability.

## 4. Conclusions

In this study, we focused on surface modification with hydrophilic material and an antibacterial agent to simultaneously improve the antifouling and antibacterial properties of the PU film. We successfully created PHMG/HA multilayer films on PU by using layer-by-layer self-assembly with COOH-activated HA and PHMG as polyanions and polycations. An optimal film named as PU-(PHMG/HA)_5_/5-5 with the lowest WCA and medium roughness was obtained, which possessed excellent protein repelling performance. The adsorption levels of BFG, HSA, and LYS reduced 67.85%, 85.33% and 80.31%, respectively, compared with that on the PU film. In addition, the high bacteriostatic rate of over 99.9% against the three tested bacteria and excellent antibacterial adhesion property showed that PU-(PHMG/HA)_5_/5-5 possessed high antimicrobial and anti-biofouling performance. Furthermore, the film had nearly no cytotoxicity against L929 cells, which made it possible for biomedical applications in the future.

## Figures and Tables

**Table 1:** The formulations of the PU-(PHMG/HA)_n_ films synthesized.

Sample Code	HA (mg/mL)	PHMG (mg/mL)	Number of Bilayers
PU-(PHMG/HA)_5_/2-2	2	2	5
PU-(PHMG/HA)_5_/5-2	5	2	5
PU-(PHMG/HA)_5_/5-5	5	5	5
PU-(PHMG/HA)_5_/5-10	5	10	5
PU-(PHMG/HA)_10_/5-5	5	5	10
PU-(PHMG/HA)_1_/5-5	5	5	1

**Table 2:** The surface roughness value of the PU-(PHMG/HA)_n_ films.

Sample Code	PU-(PHMG/HA)_1_/5-5	PU-(PHMG/HA)_5_/2-2	PU-(PHMG/HA)_5_/5-2	PU-(PHMG/HA)_5_/5-5	PU-(PHMG/HA)_5_/5-10	PU-(PHMG/HA)_10_/5-5
**RMS Roughness Value (nm)**	173.9 ± 3.3	157.0 ± 1.9	156.5 ± 1.7	136.5 ± 3.4	131.9 ± 4.2	130.8 ± 2.6

**Table 3:** Antibacterial test of films against *E. coli*, *P. aeruginosa*, and *S. aureus*.

Samples	*E. coli*		*P. aeruginosa*		*S. aureus*	
	Colonies (×10^5^, CFU/cm^2^)	Inhibition (%)	Colonies (×10^5^, CFU/cm^2^)	Inhibition (%)	Colonies (×10^5^, CFU/cm^2^)	Inhibition (%)
PU	29.2 ± 8.77	/	14.0 ± 0.283	/	24.3 ± 0.778	/
PU-PHMG	0.0345 ± 0.00127	99.88	0.0153 ± 0.00148	99.89	0.160 ± 0.0247	99.34
PU-(PHMG/HA)_5_/5-5	0	99.99	0.00514 ± 0.000247	99.96	0	99.99
